# Social Robots and Brain–Computer Interface Video Games for Dealing with Attention Deficit Hyperactivity Disorder: A Systematic Review

**DOI:** 10.3390/brainsci13081172

**Published:** 2023-08-07

**Authors:** José-Antonio Cervantes, Sonia López, Salvador Cervantes, Aribei Hernández, Heiler Duarte

**Affiliations:** Department of Computer Science and Engineering, Universidad de Guadalajara, Ameca 46600, Mexico; antonio.alvarez@academicos.udg.mx (J.-A.C.); salvador.cervantes7964@academicos.udg.mx (S.C.); ilse.hernandez9634@alumnos.udg.mx (A.H.); heiler.duarte2519@alumnos.udg.mx (H.D.)

**Keywords:** social robots, serious video games, human–robot interaction, brain–computer interface, ADHD

## Abstract

Attention deficit hyperactivity disorder (ADHD) is a neurodevelopmental disorder characterized by inattention, hyperactivity, and impulsivity that affects a large number of young people in the world. The current treatments for children living with ADHD combine different approaches, such as pharmacological, behavioral, cognitive, and psychological treatment. However, the computer science research community has been working on developing non-pharmacological treatments based on novel technologies for dealing with ADHD. For instance, social robots are physically embodied agents with some autonomy and social interaction capabilities. Nowadays, these social robots are used in therapy sessions as a mediator between therapists and children living with ADHD. Another novel technology for dealing with ADHD is serious video games based on a brain–computer interface (BCI). These BCI video games can offer cognitive and neurofeedback training to children living with ADHD. This paper presents a systematic review of the current state of the art of these two technologies. As a result of this review, we identified the maturation level of systems based on these technologies and how they have been evaluated. Additionally, we have highlighted ethical and technological challenges that must be faced to improve these recently introduced technologies in healthcare.

## 1. Introduction

Attention deficit hyperactivity disorder (ADHD) is a psychiatric neurodevelopmental disorder characterized by inappropriate levels of inattention, impulsivity, and hyperactivity [1]. ADHD is the most common mental health diagnosis in children that affects the life of a large number of young people in the world [2]. There is evidence that some children living with ADHD commonly present other conditions in addition to the symptoms of hyperactivity, impulsivity, and lack of concentration, such as a poor tolerance of frustration, low self-esteem, and mood change [3]. Even worse, when this mental condition persists into adulthood, these people present higher college dropout rates, poor job performance, difficulty keeping a job, and higher-risk impulsive behaviors such as substance abuse, self-harm, and suicide attempts [4]. Nevertheless, timely treatment in childhood can improve mental health and lifestyle during adulthood [5]. On the other hand, there are studies that show that some adolescents live pretty well with ADHD. According to [6], a group of adolescents considered ADHD a personal characteristic that comprised part of their identity. Furthermore, some adolescents reported that ADHD gave them strengths (e.g., energetic, fun, creative, outgoing, talkative, and funny), and they were happy with these aspects of their personalities, despite them also posing difficulties for them.

### Digital Technologies for Supporting Children Living with ADHD

In recent years, non-pharmacological treatments, such as behavioral interventions and cognitive training [7,8,9], have been an alternative to avoid using medications in people living with ADHD. Additionally, the computer science and mental health care research communities have been working together on developing novel technological systems for dealing with ADHD, such as BCI video games [10,11], virtual reality [12], augmented reality [13], artificial intelligence [14], robotics [15], and eye-tracking systems [16,17]. In recent years, there has been growing interest in how people living with ADHD can use social robots and BCI video games to support their health and well-being. These technologies have been considered to be implemented in therapy applications in order to make therapeutic work more attractive to children living with ADHD [18,19,20]. Therefore, robots and BCI video games are designed with playful aspects in order to strengthen the children’s intrinsic motivation and maximize the cognitive training’s positive effects through better -children’s involvement [18,20]. Regarding social robots, they have also been used as a support tool in the homework activities of children living with ADHD [21]. Thus, social robots aim to support therapists by automating the supervision, coaching, motivation, and companionship aspects of interactions with children living with ADHD.

In contrast, BCI video games are serious games characterized by implementing a brain–computer interface (BCI) to interact with the video game rather than a keyboard or joystick. BCI video games are also characterized by including the neurofeedback technique. Thus, BCI video games can help maintain the ADHD children’s motivation and attention to the task and guide them to achieve a specified goal (namely, specific changes in electrical activity in the brain) by maintaining a specific “mental state.” Studies have shown that children living with ADHD had improved behavioral and cognitive variables after frequency training (e.g., theta/beta) [10,22,23]. 

Studies reported in the literature discuss several recent large-scale projects (e.g., [22,24,25,26,27,28,29]). These projects have explored child–robot interaction and child–video game interaction to help children with developmental disorders such as ADHD. This study presents a systematic review from an interdisciplinary approach to the current state of these two technologies. The research questions used to guide this review were as follows:

- Q1: How have social robots and BCI video games been evaluated?
- Q2: Are there still open challenges in social robots and BCI video games to improve their impact and benefit on children living with ADHD?

These questions aim to provide a structured methodology for categorizing the current state of the art of these two technologies for dealing with ADHD. These questions also seek to clarify the current research limitations for future directions. The rest of this paper is structured as follows. Section 2 offers a detailed systematic review of the current state of the art regarding social robots for dealing with ADHD. Section 3 provides a systematic review of the current state of the art regarding BCI video games for dealing with ADHD. After that, Section 4 offers a discussion from an interdisciplinary approach of the results published in the literature to address the research questions. Section 5 describes the challenges with these two technologies that still need to be addressed to offer effective computational systems for dealing with ADHD. Finally, Section 6 provides some concluding remarks about the current state of systems based on social robots or BCI video games focused on supporting non-pharmacological treatments for children living with ADHD.

## 2. Study 1: Social Robots for Dealing with ADHD

### 2.1. Search Strategy

**Figure 1:**
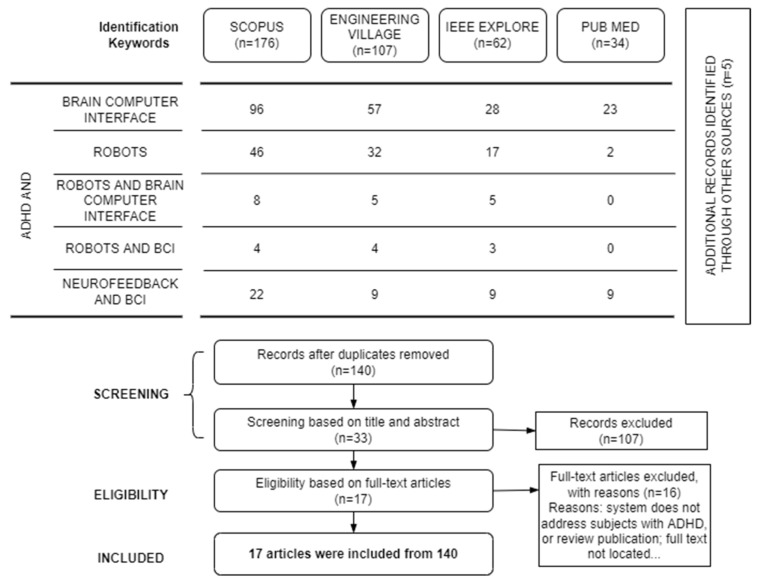
PRISMA flow diagram of robots in ADHD care.

This study was conducted through a systematic literature review called the Preferred Reporting Items for Systematic reviews and Meta-Analyses (PRISMA) [30]. Papers were sought from four major online databases: PubMed to provide a medical perspective, Engineering Village and IEEE Xplore to provide an engineering perspective, and Scopus to provide a cross-disciplinary perspective. The search was limited to papers published from January 2010 to 15 June 2022. Figure 1 shows the search strategy and keywords used to search for papers on PubMed, Engineering Village, IEEE Xplore, and Scopus.

### 2.2. Eligibility Criteria

The following inclusion criteria were considered: (1) papers that have been published in international journals or international congresses; (2) papers written in English; and (3) papers that described the conceptualization, development, testing, or evaluation of robots for use with people living with ADHD. Additionally, the following exclusion criteria were considered: (1) papers dealing exclusively with ADHD, (2) papers on robots for use with people without identified health conditions, and (3) review papers.

### 2.3. Screening

The screening process was divided into two phases (see Figure 1). The first phase involved removing duplicate records. After that, two authors independently screened the titles and abstracts to remove those papers that did not meet the eligibility criteria. In the second phase, the same two authors obtained and screened full texts for the remaining papers. Any differences were resolved through consulting with a third author.

### 2.4. Search Results

The initial search for social robots in ADHD care was conducted with different combinations of word keys (see Figure 1), producing 332 results. Once duplicates were removed, 140 publications remained. The screening of titles and abstracts resulted in a working pool of 33 publications. Full-text articles were thoroughly screened according to the eligibility criteria, resulting in 17 publications that were considered for studying and then describing the recent advances and challenges in systems based on social robots for dealing with ADHD. Of the 17 papers included, 52.94% (9/17) were papers published in a journal, and 47.06% (8/17) were papers published at an international conference.

Table 1 shows how each paper was sorted regarding the target population and proposed application type. Thus, 94.12% of the papers found in the literature focused on social robots for children living with ADHD, and just 5.88% focused on social robots for undergraduate students living with ADHD. According to the papers’ proposed aims and application types, we identified one paper that presents a methodology for designing robot-assisted therapy [24]. This methodology is characterized by proposing an iterative approach where patients, parents, and therapists take the role of stakeholders to ensure a customized implementation of the technology that meets patients’ specific needs. On the other hand, two of the reviewed papers propose a novel system for supporting the diagnosis of ADHD [25,31], whereas in [25], the aim was to investigate the clinical effectiveness of a new robot-assisted kinematic measure for ADHD. In [31], a robot-assisted test for measuring ADHD symptoms in children is presented. The rest of the reviewed papers propose a novel system supporting rehabilitation therapies for children living with ADHD. Also, we found that some systems have been designed for children living with ADHD who have an additional developmental disability, such as autism spectrum disorder (ASD) [18,24,32,33], oppositional defiant disorder (ODD) [27], cerebral palsy (CP) [34], and learning disability [33]. Additionally, Table 1 shows the type of social robot implemented in each proposed system and the input signal or sensors used to assess the children’s behavior. Thus, eleven systems implemented humanoid robots like Nao [18,24,32,35], Silbot [25,31], Robotis Bioloid [36], Pepper [19], Sanbot Elf [37,38], and Ifbot [39], and just six systems implemented a social robot developed ad hoc [21,27,33,34,40,41]. Finally, we highlighted the type of environment used in each system. It could be virtual, real, or hybrid. This last type means that participants interact with a physical robot, but they also interact with software commonly hosted on a secondary device such as a desk computer, laptop, or tablet. Ten systems are based on a real environment, and seven are based on a hybrid one. None of the systems identified in the literature are based on a purely virtual environment.

Table 2 summarizes the results reported in the literature on developing social robots for dealing with ADHD. In total, 16 of 17 papers described in this review presented a study. According to the characteristics of each study, we classified them as feasibility, effectiveness, and usability studies. A total of 56% of studies reported in the literature were considered feasibility studies. These studies focused on testing the proposed system’s potential for helping children living with ADHD. In total, 25% of the studies were classified as usability studies. These studies focused on evaluating the satisfaction and acceptance level of the proposed system. Finally, just 19% of studies were classified as effectiveness studies. These studies investigated the clinical effectiveness of robot-assisted therapy on children living with ADHD. Additionally, we identified that the reported studies in the literature involved people with different characteristics. For instance, two studies involved just healthy children [35,41], one study involved undiagnosed children with potential symptoms of ADHD [21,25,31,36], six studies involved children living with ADHD and other developmental disorders [18,24,27,32,34,38], and three studies involved just children living with ADHD [19,37,40]. Table 2 shows the participants’ characteristics in each study case, such as the number of participants, age, gender, and health conditions.

Moreover, we identified that the duration of these studies was very variable. For example, some studies were based on a few sessions (e.g., four sessions) and involved fewer than ten participants in the trials (e.g., [19,37]), whereas other studies were based on many sessions (e.g., multiple sessions during a month or more) with more than ten children (e.g., [25,27,31,32]). We observed that most of these studies were conducted in collaboration with experts in the healthcare area. The last column of Table 2 summarizes the results obtained in each study. 

According to the results reported in the literature, we consider that systems based on social robots for dealing with ADHD are promising technology. However, the use of social robots for diagnosing or treating children living with ADHD is still in its first phases of development. Also, based on the number of participants involved and the duration of the trials reported in the literature, we have considered that the results reported in these studies can be regarded as preliminary results. Therefore, more trials with more significant populations are needed to validate such results.

## 3. Study 2: BCI Video Games for Dealing with ADHD

### 3.1. Search Strategy

**Figure 2:**
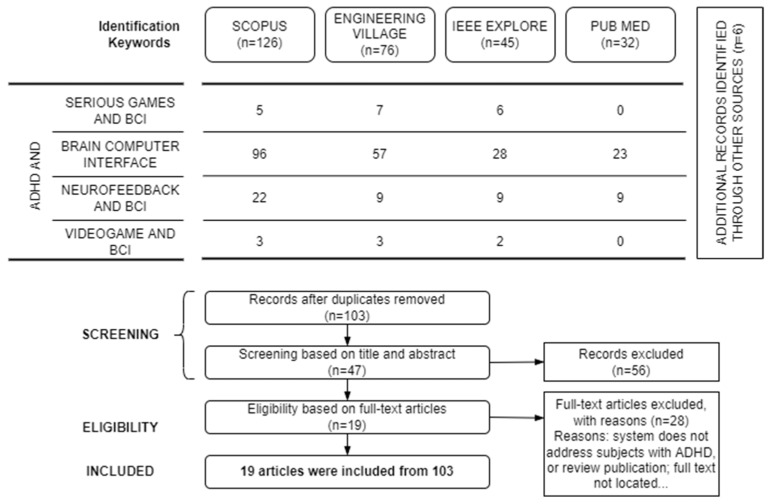
PRISMA flow diagram of BCI video games in ADHD care.

This second study used the same strategy implemented in the previous study of social robots. Therefore, papers were sought from four major online databases: PubMed to provide a medical perspective, Engineering Village and IEEE Xplore to provide an engineering perspective, and Scopus to provide a cross-disciplinary perspective. The search was limited to papers published from January 2010 to 15 June 2022. The keywords used for searching papers were “ADHD” AND “serious games” AND “BCI”, “ADHD” AND “brain-computer interface,” “ADHD” AND “neurofeedback” AND “BCI”, “ADHD” AND “videogame” AND “BCI”. These keywords were adapted according to the user interface offered by each online database. Figure 2 shows the search strategy used in this study.

### 3.2. Eligibility Criteria

Publications were included if they were published in international journals or international congresses, were written in English, and described BCI video games’ conceptualization, development, testing, or evaluation for people living with ADHD. On the other hand, publications on ADHD exclusively and BCI video games for use with people without identified health conditions, as well as review papers, were excluded.

### 3.3. Screening

The screening process was divided into two phases (see Figure 2). The first phase involved removing duplicate records. After that, two authors independently screened the titles and abstracts to remove those papers that did not meet the eligibility criteria. In the second phase, the same two authors obtained and screened full texts for the remaining papers. Finally, any discrepancy was resolved through consulting with a third author.

### 3.4. Search Results

After the screening and eligibility criteria were applied, 19 papers from the initial 103 candidates remained for review. The selected papers were utilized to investigate the features of systems based on BCI video games for dealing with ADHD. Afterward, the characteristics of each system, such as the advantages and disadvantages, experimental environment, and applications, were discussed. Figure 2 shows the flow diagram of PRISMA with the results obtained after applying the keywords in the queries.

Table 3 summarizes BCI video game projects reported in the literature. These research projects focus on the cognitive training of both healthy people and people living with ADHD. BCI video games commonly use the neurofeedback approach. Therefore, the primary input signal of BCI video games comes from the human brain’s bioelectrical activity. Thus, these systems seek to offer an alternative or complement to traditional pharmacological treatments. BCI video games aim to improve different cognitive capabilities, such as attention, concentration, spatial memory, short and long memory, and motor skills (e.g., [28,42,43,44]). These systems are commonly designed using a playful approach where the BCI video game offers challenges, missions, goals, and different complexity levels to catch the participants’ interest and attention.

A total of 14 of the 19 projects described in Table 3 were designed to support the children’s cognitive training, whereas the remaining projects included in Table 3 did not consider the participants’ age for their design. Additionally, we observed that BCI video games offer a wide variety of themes such as farms, racing, spaceships, forest, classroom, puzzle, mazes, and under the ocean. However, research questions may arise for future research, such as whether this kind of video game may generate a type of addiction and how skills obtained through playing these BCI video games can be applied in daily life.

Table 4 describes trials and the results of each study presented in Table 3. Also, the main characteristics of participants involved in trials, such as the number of participants, their age, gender, and health conditions, are included in Table 4. We identified that 53% of the works reported in the literature focused on studying the effectiveness of systems based on BCI video games, 32% focused on studying the feasibility of using these systems on children living with ADHD, 11% focused on researching whether changes occurred at an anatomical brain level in children living with ADHD after using a BCI video game, and only 5% focused on testing the usability of BCI video games on children living with ADHD.

Three types of trials were identified in the effectiveness studies: (i) trials that just involved an experimental group formed by children living with ADHD [44,51], (ii) trials similar to the previous but with healthy people [43,55], and (iii) trials that involved children living with ADHD who were divided into experimental and control groups to compare the results obtained with both groups [10,22,23,28,49,50]. Additionally, we observed that 50% of these studies involved more than 50 children living with ADHD, whereas the rest involved between 20 and 26 children living with ADHD. Furthermore, we observed that most feasibility studies involved healthy participants instead of participants living with ADHD. This is because the systems proposed in these papers are still in the early stages of development.

## 4. Discussion

This systematic review of social robots and BCI video games was conducted from an interdisciplinary approach. Therefore, the results reported in this paper focused on highlighting relevant information in engineering and healthcare areas. For instance, Table 1 and Table 3 show useful information for the engineering area. This information is related to the type of application developed in each paper. We grouped applications into three groups. The first group includes applications for supporting rehabilitation therapies. Most applications identified in the literature belong to this group. A second group of applications focused on supporting ADHD diagnosis. An application in this group claims to have reached 97% confidence in identifying children living with ADHD [31]. The last group involves applications focused on supporting neuroscience research. Additionally, Table 1 and Table 3 present the aim of each paper, such as proposing a methodology for designing social robots or BCI video games, presenting the architectural design of these systems, and comparing their performance, among other objectives.

Concerning the robot-based applications, we identified that the most common robots used in these applications are humanoid robots such as Nao, Silbot, Robotis Bioloid, Pepper, Sanbot Elf, and Ifbot. We also highlighted input signals and environments used in such applications (real, virtual, and hybrid). For this last characteristic, we identified that the real environment has been used most in these applications. Regarding BCI-based video game applications, we identified that the environments more used (listed in order of preference) are 3D, 2D, virtual reality, and mixed reality environments.

Although most papers in the literature were published in engineering journals or proceedings, we could identify relevant information about these two technologies for the healthcare area. Table 2 and Table 4 describe the main characteristics of participants involved in trials, such as the number of participants, their gender, age, and health conditions. Additionally, these tables offer a brief description of the results reported in each study, including the acceptability level of technology. Also, we tried to identify whether these technologies can significantly reduce some symptoms associated with ADHD or improve any skill ability. We observed that systems based on social robots or BCI video games are designed mainly to reduce attention deficit and improve some skills, such as learning, writing, spatial memory, working memory, communication, and interaction.

This systematic review lets us answer our two research questions. Most systems reported in the literature are still in the developing and testing phases, and just two applications named Focus Pocus [10] and EndeavorRx [56] are commercialized and prescribed for treating children living with ADHD. In total, 88% of robot-based applications found in the literature have been tested involving people living with ADHD. Furthermore, some of these applications have involved people living with ADHD who have an additional developmental disability, such as ASD [18,24,32,33], ODD [27], CP [34], and learning disability [33]. These applications have been tested for different purposes, such as validating their functionality and exhibiting their potential benefits on people living with ADHD.

On the other hand, BCI video game systems have been evaluated in their functionality and usability by including healthy participants [29,45,47,48] and participants living with different levels of ADHD [54]. This kind of system has been compared against the most used neurofeedback system (cartoon-based). The results published in [28] suggest that BCI video games can be better than cartoon-based systems for maintaining people’s attention. These comparisons also include medicine-based treatments, where BCI video game systems showed similar results in the reduction in ADHD symptoms, especially in the inattentive symptoms [22,46,50]. Additionally, evaluations with healthy individuals revealed that the BCI video game systems could improve attention levels in healthy and ADHD individuals [43,54].

Finally, we identified a set of challenges that developers must face for developing flexible systems that can be reconfigured and customized according to the behavior and deficit of each individual. These challenges are described in detail in the following section.

## 5. Open Challenges

This section describes the challenges identified during the systematic literature review for developing computational systems based on social robots or BCI video games to treat ADHD. These challenges are related to the system’s target, the design of a general system for dealing with all types of ADHD, the problem of customizing training exercises, and some ethical issues, such as making sure not to compromise the children’s social–emotional development.

- Diagnosing ADHD. The symptoms presented by a child living with ADHD can differ from those of another child living with ADHD. This is because there are three different subtypes of ADHD [57]: predominantly inattentive type (ADHD-I), predominantly hyperactive/impulsive type (ADHD-HI), and combined type (ADHD-C). To complicate matters even more, there are people living with ADHD who have an additional neurodevelopmental disorder, such as ASD [24,28], ODD [27], and anxiety problems [27]. Therefore, developing a general system for diagnosing people with diverse forms of ADHD represents a hard challenge.
- Customizing cognitive training exercises. Customizing cognitive training exercises according to the characteristics of each person is another hard challenge for engineers and researchers involved in designing general systems based on robots and BCI video games for dealing with ADHD [18,24,32]. This is due to different factors, such as the subtype of ADHD [57], the level of ADHD (from moderate to severe) [58], the preferences of each person [18,32], and poor adaptation to the users’ level of expertise with the technology leading to frustration [59], among other characteristics associated with the neurodevelopment of children living with ADHD, which should be considered in order to offer people a great experience.
- Capturing and maintaining attention. Engineers and researchers agree that one complex challenge when designing a cognitive training system is creating one capable of capturing and maintaining children’s attention in each cognitive training session [55,57]. The findings published in the literature suggest that children’s enjoyment and engagement decline across cognitive training sessions because exercises offered by those systems become routine and repetitive after several sessions [10].
- Long-term or longitudinal studies. Most systems reported in the literature indicate that they can be useful tools for diagnosing or treating ADHD, according to their objective (e.g., [18,23,25,27,51]). However, most systems have been tested with small groups of children (fewer than 20 participants). These studies are also characterized by considering few cognitive training or therapeutic sessions. Testing these systems on children living with ADHD is not a trivial problem. The first difficulty is related to recruiting children living with ADHD, and the second is associated with parents’ and children’s interest and perseverance in attending all sessions. According to Baxter et al. [60], only 5 out of 96 empirical studies in the human–robot interaction field consisted of more than a single session between 2013 and 2015. Therefore, long-term or longitudinal studies must be conducted to document changes over time, which can provide more reliable and accurate information about the efficacy of these systems focused on dealing with ADHD.
- Certification. The little evidence reported in the literature suggests that systems based on social robots or BCI video games with neurofeedback have great potential to improve the attention of children living with ADHD (e.g., [10,18,22,25,45]). Nowadays, few systems have been approved to be prescribed for treating children living with ADHD (e.g., [10,56]). Therefore, obtaining approval from a certification entity represents a challenge because all these systems are relatively new and most are currently in the design and prototyping phases.
- Avoiding physical harm. Some characteristics of robots can represent limitations and challenges for designing a safe human–robot interaction. Therefore, researchers and engineers should carefully choose the type of robot to use in a cognitive training system and define how these robots can interact with people involved in a cognitive training session to avoid potential physical harm. A good example of this issue can be observed in the system proposed by Cervantes et al. [20], called CogniDron-EEG. This system includes a drone. Therefore, using a drone implies avoiding physical interaction such as touching it. Even more, flying a drone at a reachable distance and in a small room can represent a potential issue of human–robot interaction if safety control mechanisms are not implemented appropriately.
- Making sure not to compromise social–emotional development. Few researchers are worried about how social robots can compromise children’s social–emotional development [61]. Children living with ADHD or other mental disorders can be more sensitive than regular children to socially affective bonding with a robot. According to Sandygulova et al. [24], there is a potential risk that individuals might develop strong emotional bonds with the robot, the severing of which at the end of therapy can have negative effects on individuals, such as a recoil in therapeutic benefits that the person might have achieved. However, there are few studies related to this ethical issue because, given the current state of this technology, it seems to be more urgent to address other ethical issues and challenges, such as privacy, safety, and the efficacy of these systems in dealing with ADHD.

## 6. Conclusions

The systematic review conducted in this paper allows us to know the current state of systems based on social robots and BCI video games for dealing with ADHD. We found 36 systems; 17 are based on social robots, 18 are based on BCI video games, and 1 is based on serious video games without including a BCI. According to the functionality of these systems, we have classified them into three types: systems for diagnosis, systems for cognitive training, and systems for studying the human brain. Of all the systems identified in the literature, we observed that 2 systems focused on supporting the diagnosis of ADHD in people, 32 systems focused on supporting cognitive or behavioral rehabilitation therapies, and 2 systems focused on supporting the study of brain areas of people living with ADHD. The results reported in the literature have shown the potential of these systems for supporting non-pharmacological treatments of people living with ADHD or detecting ADHD in people. However, the research community focused on these technologies agrees that more studies must be conducted in order to identify both the advantages and disadvantages of using these systems in real clinical environments. For instance, some studies have reported positive effects on children living with ADHD when they interact with these technologies. Some of these positive effects are a quick acceptance of technology, better engagement during therapy sessions, positive experiences using these novel technological systems, and relevant improvement in the cognitive functions of people who have been trained through these systems. Nevertheless, researchers have also found that a small group of children has had bad experiences or a kind of frustration when using these new technologies. These negative findings may indicate that not all people living with ADHD can be candidates for using these technologies. As a result of this review, we identified a set of critical challenges that developers must face when developing flexible systems that can be reconfigured and customized according to the characteristics of each individual, in order to offer the best experience to the patient. Finally, most systems reported in the literature are still developing and testing processes. We hope this study can help guide the development of future systems based on social robots or BCI video games for children living with ADHD.

## Figures and Tables

**Table 1:** The current state of social robots for dealing with ADHD.

Source	The Aim of the Paper	Application Type	Target Population	Type of Robot	Input Signal	Environment
[24]	To propose a methodology for designing robot-assisted therapy. Additionally, this paper shows how to use the methodology by designing and implementing a robotic application.	Methodology for designing robot-assisted therapy	Children living with a diverse form of ASD combined with ADHD	A humanoid Nao	Tactile sensors	Real
[25]	To study the clinical effectiveness of a robot-assisted kinematic measure of ADHD.	Support for ADHD diagnosis	Children living with ADHD	A humanoid Silbot	A 3D camera, LED sensors, and a 3D depth sensor	Real
[27]	To address the issue of standardizing and automatizing therapies for children living with ADHD and ASD.	Support for rehabilitation therapies for children living with ADHD and ASD	Children living with ADHD, ASD, ODD, and anxiety problems	A caretaker Robot (CARBO)	Tactile sensors	Hybrid
[35]	To present an architectural design of Kindergarten Assistive Robotics (KAR) focused on children living with ADHD.	Support for rehabilitation therapies	Kindergarten children living with ADHD	A humanoid Nao	Cameras, microphones, ultrasonic sensors, and tactile sensors	Real
[32]	To present ongoing work that aims to target children with ASD and ADHD. Additionally, this paper describes a novel behavior used to introduce and practice a set of social behaviors.	Support for rehabilitation therapies	Children living with ASD and ADHD	A humanoid Nao	The paper does not indicate which of Nao’s sensors were used	Real
[18]	To conduct a long-term study using social robots to create an individualized experience in robot-assisted therapy for children with diverse forms of autism and ADHD to bring positive changes in their behaviors through long-term engagement.	Support for rehabilitation therapies	Children living with ASD and ADHD	A humanoid Nao	Tactile sensors	Real
[34]	To explore the efficacy of robotic technology in improving handwriting in children with poor motor skills.	Support for rehabilitation therapies	Children living with cerebral palsy (CP), children living with ASD, children living with ADHD, and children living with other disorders that affect good motor skills	A robot based on a haptic device	No input signals	Real
[39]	To study the effects of collaborative learning between robots and children with developmental disabilities.	Support for rehabilitation therapies	Children living with potential symptoms of a developmental disability	An Ifbot robot	No input signals	Real
[36]	To propose a cognitive architecture for improving the interaction between a humanoid robot and preschool children living with ADHD using joint attention during turn-taking gameplay.	Support for rehabilitation therapies	Children living with ADHD	A humanoid Robotis Bioloid	Several peripheral body sensors (e.g., a 2-axis gyro, joint position encoders, IR transmitter, and a proximity sensor for distance measurement) and sensors belonging to Microsoft’s Xbox Kinect (e.g., camera, depth sensor, and a multi-array microphone)	Real
[40]	To present the design and initial evaluation of a social robotic device that provides immediate feedback for students living with ADHD.	Support for rehabilitation therapies	Students living with ADHD	A Kip3 robot	Through a test presented on a tablet	Hybrid
[19]	To propose a therapeutic methodology based on human–robot interaction for improving the social skills of children living with ADHD.	Support for rehabilitation therapies	Children living with ADHD	A humanoid Pepper	A touchscreen and two cameras	Hybrid
[37]	To present a system’s design, development, implementation, and assessment to remotely control a robot for rehabilitating children living with ADHD.	Support for rehabilitation therapies	Children living with ADHD	A humanoid Sanbot Elf and augmented reality smart glasses	EEG	Hybrid
[41]	To present the design, development, implementation, and assessment of an intelligent home environment.	Support for rehabilitation therapies	Healthy children and children living with attention disabilities (including children living with ADHD)	A small robot named Atent@	A touchscreen and a proximity sensor were placed on the robot, an accelerometer sensor was placed on a chair, and a proximity sensor was placed on a desk	Hybrid
[21]	To present the design, development, implementation, and assessment of a robot assistant.	Support for rehabilitation therapies	Children living with ADHD	A small robot named Atent@	A touchscreen, proximity sensors, and an accelerometer sensor	Hybrid
[38]	To assess a wearable BCI based on augmented reality to remotely control a robot for rehabilitating children living with ADHD.	Support for rehabilitation therapies	Children living with ADHD	A humanoid Sanbot Elf and augmented reality smart glasses	EEG	Hybrid
[33]	To present the mechanical design and performance of a robot designed for supporting children with developmental disorders.	Support for rehabilitation therapies	Children living with ASD, ADHD, and learning disabilities	A small robot in the form of a sphere	No input signals	Real
[31]	To present a novel approach to screening childhood ADHD using robotic and machine learning technologies.	Support for ADHD diagnosis	Children living with ADHD	A humanoid Silbot	An RGB-D sensor	Real

**Table 2:** Studies and results of using social robots for dealing with ADHD.

Article	Literature	Type of Study	Participants	General Results	Acceptability	Near and Far Transfer Effects
[24]	Engineering journal	Usability study	14 children (5 boys and 1 girl) aged 3 to 8 years old, ±1.46, with a mean age of 5.28 years old. All these children have been diagnosed with ASD and ADHD.	A methodology for designing a robot-assisted therapy was proposed. Additionally, this methodology was applied for designing appropriate robot behaviors tailored for the diverse forms of ASD and ADHD in children. After that, a trial with 6 children was conducted. The result of this trial suggests that most children enjoyed the interaction with the robot. However, the effectiveness of this methodology is still being studied due to the small sample size involved in the controlled trial.	86% of children enjoyed interactions with the robot.	86% of children showed a slight improvement in maintaining their attention during training sessions and enhanced their tactile interaction with the robot.
[25]	Medical journal	Effectiveness study	35 children living with ADHD aged 5 to 12 years old (30 males and 5 females, ±1.5, mean age = 8.8) and 50 healthy children as control (23 males and 27 females, ±0.9, mean age = 8.7).	According to [25], differences between the ADHD and healthy control groups were observed regarding most variables of the robot-assisted kinematic measure for ADHD (RAKMA), including correct reactions, commission errors, omission errors, reaction times, migration distance, and migration speed scores. Therefore, the authors of this research claim that the RAKMA is a clinically useful tool for objectively measuring hyperactivity symptoms in children living with ADHD.	No available information.	Target out of scope.
[27]	Engineering proceedings	Feasibility study	18 children between 7 and 11 years old. Children were grouped according to their developmental disorder: 5 children had only ADHD, 4 children had ADHD and ODD, 3 children had ADHD and anxiety, 5 children had ADHS and ASD, and 1 child had only ASD.	Preliminary results of this study show the robot’s potential as a diagnostic tool for children with neurodevelopmental problems. However, the authors also indicate that more extensive studies must be conducted to confirm their preliminary results.	100% of children enjoyed interactions with the robot because they considered that interaction with the robot was exciting and intuitive.	Target out of scope.
[35]	Engineering proceedings	Feasibility study	18 healthy children, aged 4 to 8 years old, half boys and half girls.	Results reported by [35] indicate that most of the children involved in the trial were able to create a positive interaction with the robot, except 2 children. However, this study did not involve children living with ADHD to know whether it could be useful in dealing the ADHD in children.	89% of children showed a positive interaction.	Target out of scope.
[32]	Engineering proceedings	Feasibility study	15 children (all males) aged 3 to 12 years old, ±2.7, with a mean age of 6.7 years old. All children have been diagnosed with ASD and ADHD.	Preliminary results reported by [32] indicate that the robot established a satisfactory level of engagement during the robot-assisted therapy sessions. Also, the authors affirm that all parents noted improvement in their children’s social skills, such as eye contact and concentration.	100% of children showed a positive acceptance of working with the robot.	100% of children showed a significant improvement in sustained attention and eye contact. Additionally, several nonverbal children started to pronounce simple words, such as “Bye,” “Tick-Tack,” and “Nao”.
[18]	Engineering journal	Effectiveness study	11 children (1 girl and 10 boys) aged 4 to 11 years old, ±2.7. 4 children were diagnosed with ASD, while 7 were diagnosed with ASD and ADHD.	This paper presents a quantitative analysis of a multiple-session study conducted with children with ASD and ADHD. The findings from this study suggest that (i) it is possible to sustain engagement in children with autism and/or ADHD when they interact with a robot over multiple sessions and (ii) children are better engaged and focused during robot-mediated sessions when activities are responsive to each child’s preferences and likes.	100% of children accepted working with the robot.	100% of children maintained their engagement and eye gaze on their activities in all sessions.
[34]	Medical journal	Effectiveness study	18 children (14 boys and 4 girls) aged 5 to 11 years old. 6 children with no reported disability were referred to the study because of plodding handwriting speed, 2 children living with ADHD, 5 children with ASD, 1 child with pervasive developmental delay, 2 children with intellectual disability, and 2 children with deafness.	Results reported in this study indicate that fine motor control improved for children with learning disabilities and those aged 9 or older but not for those with CP or under age 9. Also, all children with ASD or ADHD referred for slow writing speed were able to increase speed while maintaining legibility.	89% of children found the robot very engaging.	Therapy allows children to concentrate on the shape and size of letters. 100% of children with ASD or ADHD referred for slow writing speed were able to increase speed while maintaining legibility.
[39]	Engineering proceedings	Feasibility study	3 undiagnosed children who had potential symptoms of ADHD.	The results of this study suggest that the robot prompts children to improve their concentration while collaboratively learning. Additionally, researchers found that the learning time during the collaborative learning session was greater in the robot’s presence than without the robot.	100% of children accepted working with the robot.	100% of children showed significatively increased attention and learning time when the robot participated in the learning sessions.
[36]	Engineering journal	Feasibility study	A normal group of children and a group of children diagnosed with ADHD.	The results reported in this study revealed an increase in sustained attention and a decrease in response time as interaction scores increased. Additionally, the results showed a gradual decrease in the differences in interaction scores and reaction time performance between the normal and ADHD groups.	100% of children in both groups accepted working with the robot.	A slightly increased sustained attention was observed in all children in both groups. Additionally, all children worked on their emotional responses and episodic memory.
[40]	Engineering proceedings	Usability study	10 undergraduate students were recruited for the study, all diagnosed with ADHD, aged 20 to 35 years old, ±3.43, with a mean age of 26.3 years old. 4 males and 6 females.	In this study, 9 participants reported a positive experience. These participants considered that the Kip3 robot helped them regain focus on a task because of the real-time feedback on their performance. Only one participant indicated that the real-time feedback was not a positive experience because the participant felt frustrated by the feedback signal.	90% of children enjoyed working with the robot.	90% of children were able to regain attention during training sessions.
[19]	Engineering proceedings	Usability study	5 children living with ADHD, aged 7 to 10 years old.	The goal of this study was to evaluate the degree of acceptance of the introduced technology support. The results of trials indicated that children immediately accepted the presence of a humanoid robot. In fact, children were starting to collaborate and showing a higher degree of attention than in the traditional therapy exercise (without a robot) they had to perform.	100% of children immediately accepted working with a robot from their first session.	All children showed better attention regarding the exercise they had to perform when the robot was introduced in their therapy sessions.
[37]	Engineering journal	Feasibility study	4 children living with ADHD, aged 6 to 8 years old.	The results of this study offered feedback on the wearability and usability of the device. Additionally, this study provided information on the children’s engagement and attentional performance.	100% of children accepted to use the novel technology.	The attention performance exhibited by all participants was far superior to that shown in traditional sessions.
[41]	Engineering journal	Feasibility study	10 healthy children aged 6 years old (5 boys and 5 girls).	This study aimed to validate the requirements provided by therapists. Additionally, this study showed the possibilities and functionalities to stakeholders and families so that they would allow the next validation phase involving children living with ADHD.	100% of children accepted the presence of the robot and valued it as a positive aid.	Target out of scope.
[21]	Engineering journal	Feasibility study	4 children (2 boys and 2 girls) of the same age (6 years old). A boy and a girl with suspected ADHD, and the other 2 children were diagnosed as healthy participants.	This study helps to validate the robot’s functionality. According to [21], the robot was able to obtain relevant information such as the time of completion of each task, number of distractions, pauses between tasks, calls for assistance, frequency of impulsivity, frequency of hyperactivity, number of completed tasks, change in mood, emission of sounds, and times that participants follow the instructions.	100% of children accepted the presence of the robot.	All children (with suspected ADHD or not) showed a reduction in their level of distraction. However, this distraction reduction was higher in healthy children (this result was already expected by the expert involved in the test).
[38]	Engineering proceedings	Usability study	18 children aged 5 to 10 years old (±1.39, mean age = 1.35). All children had different diagnoses, always including ADHD.	The results reported in this study showed that all the children between 8 and 10 years old involved in the trials completed the activities. These children indicated they felt delighted with the experience. However, some children aged 5 to 7 years old had issues related to the device’s ergonomics, and in some cases, they could not pay attention during the trial explanation.	67% of children enjoyed interacting with the technology, and the rest had issues related to the device’s ergonomics.	Target out of scope.
[31]	Engineering journal	Feasibility study	326 children from the 3rd and 4th grades of elementary school, of whom 35 were clinically diagnosed with ADHD by doctors. Another 26 children were identified as at risk for ADHD by standard tests for ADHD diagnosis.	The results reported in this study indicate that, compared to conventional questionnaire-based tests, using robotic and machine learning technologies significantly increased the accuracy of ADHD diagnosis to 97%. Also, this study allowed researchers to identify some key features of the robot (e.g., classification algorithms and optimal parameters) to classify children into three diagnostic categories of childhood ADHD: ADHD, ADHD-at-risk, and normal.	No available information.	Target out of scope.

**Table 3:** The current state of BCI video games for dealing with ADHD.

Source	The Aim of the Paper	Application Type	Target Population	Input Signal	Environment
[45]	To develop a BCI video game for enhancing the attention of children living with ADHD by presenting a realistic environment with distractors and incremental complexity	Support for rehabilitation therapies	Children living with ADHD	EEG	A 2D/3D single-player video game with incremental complexity
[28]	To compare BCI video-game-based therapy with neurofeedback and traditional therapy to treat ADHD	Support for rehabilitation therapies	Children living with ADHD	EEG	A 2D single-player video game
[22]	To compare BCI-based attention training video game with a control group in the improvement of inattentive symptoms in children living with ADHD	Support for rehabilitation therapies	Children living with ADHD	EEG	A 3D single-player video game
[46]	To investigate the brain network organizational changes in children living with ADHD during 8 weeks of BCI-based training for behavior improvement	Support for neuroscience research	Children living with ADHD	EEG/MRI	A 3D single-player video game
[42]	To propose a BCI video game with EEG to improve attention and short- and long-term memory in children living with ADHD	Support for rehabilitation therapies	Children living with ADHD	EEG	A 3D single-player video game with incremental complexity
[47]	To develop a BCI video game to observe the mental conditions of people living with ADHD for attention training and rehabilitation	Support for rehabilitation therapies	People living with ADHD	EGG	A VR 3D single-player video game
[48]	To develop a BCI system made up of 2 video games for enhancing the attention level of people living with ADHD by reading the P300 potential and providing feedback	Support for rehabilitation therapies	People living with ADHD	EGG	A VR 3D single-player video game with simulated distractions
[43]	To develop a BCI video game with different levels of complexity designed to improve cognitive skills such as attention level, mediation level, and spatial memory	Support for rehabilitation therapies	People living with ADHD	EEG	A 2D single-player video game with incremental complexity
[44]	To develop a BCI system based on video games for training sustained attention in children living with ADHD	Support for rehabilitation therapies	Children living with ADHD	EEG	A 3D single-player system with multiple video games
[49]	To evaluate the effectiveness of neurofeedback on training cognitive functions in children living with ADHD	Support for neurofeedback training	Children living with ADHD	EEG	A single-player video game based on playing a movie
[50]	To investigate the effectiveness of BCI video games with increasing difficulty in the treatment of children living with ADHD	Support for rehabilitation therapies	Children living with ADHD	EEG	A 2D single-player video game with incremental complexity
[10]	To examine the efficacy of combined working memory, inhibitory control, and neurofeedback training in children living with ADHD and subclinical ADHD	Support for rehabilitation therapies	Children living with ADHD and subclinical ADHD	EEG	A 2D video game with a single player
[51]	To present a game-based training system designed for analyzing and improving the reading ability of children living with ADHD	Support for rehabilitation therapies	Children living with ADHD in the first or second grade	EEG	A 2D video game with a single player
[52]	To investigate the effects of using a custom-made neurofeedback video game	Support for rehabilitation therapies	Children living with ADHD	EEG	A 3D video game with a single player
[53]	To investigate the relation between dopamine and reward signals on the anterior cingulate cortex in children living with ADHD	Support for neuroscience research	Children living with ADHD	EEG	A 3D video game with a single player
[54]	To present the design and development of a video game focused on promoting behavioral learning and prosocial skills in children living with ADHD	Support for rehabilitation therapies	Children living with ADHD	EEG/Keyboard	A 2D and 3D video game with a single player
[29]	To integrate a BCI with a serious video game for training and strengthening patients’ attention ability while their attention levels are monitored	Support for rehabilitation therapies	People living with ADHD	EEG	A 3D video game with a single player
[23]	To analyze the effectiveness of a game-based system for assisting children in managing and overcoming ADHD	Support for rehabilitation therapies and children living with ADHD	Children living with ADHD	Touch screen	A virtual 3D world and a multisensory mixed reality with a single player
[55]	To study the impact of a neurofeedback-based BCI game on enhancing attention and cognition skills in healthy people	Support for neurofeedback training	Healthy people	EEG	A 2D video game with a single player

**Table 4:** Studies and results of using BCI video games for dealing with ADHD.

Article	Literature	Type of Study	Participants	Results	Near and Far Transfer Effects
[45]	Engineering proceedings	Feasibility study	11 healthy participants (8 males and 3 females in the age range 27.5, ±4.5).	Results reported in this study indicate that the participants’ accuracy in a cognitive training task falls and the time increases as they advance in the BCI video game’s levels or when more distractors appear in the virtual environment. However, this study also indicates that participants can achieve the same accuracy at both basic and complex levels after they become accustomed to the BCI video game.	Target out of scope.
[28]	Multidisciplinary journal	Effectiveness study	26 children living with ADHD (age range 8, ±3.05). 13 children were randomly assigned to the experimental group, and the rest were assigned to the control group.	Children with video-game-based therapy showed a slightly more significant improvement in attention, sustained attention, and attentional control than children committed to traditional therapy (cartoon-based).	The BCI game participants showed an improvement in their attention level and were less dispersed.
[22]	Medical journal	Effectiveness study	172 children living with ADHD (147 males and 25 females in the age range 8.6, ±1.51).	After 8 weeks of therapy, both video-game-based and control groups reduced their clinical inattentive symptoms on the ADHD Rating Scale by 3.5 (±3.97) and 1.9 (±4.42), respectively. During the trials, it was reported that 11 children experienced mild to moderate adverse events of headache, dizziness, motor restlessness, and attention problems (the first two problems were the main ones). Only on one occasion did one child experience two problems at the same time (headache and attention problems).	The BCI group showed a higher reduction in inattentive symptoms than the control group.
[46]	Medical journal	Neuroscience study	66 children living with ADHD. 44 children were included in a BCI-based training and 22 children were included in a control group.	Findings reported in this study indicate that the BCI-based training group shows a more significant reorganization of the brain functional network from more regular to more random configuration than the control group.	The BCI group significantly reduced inattention symptoms more than the control group. Additionally, the BCI group showed differential brain network reorganizations after training.
[47]	Engineering proceedings	Feasibility study	10 healthy participants (age range 19 to 40).	As a result of this work, a BCI video game capable of measuring and interpreting a person’s attention-related EEG signals was developed.	Target out of scope.
[48]	Engineering in medicine proceedings	Feasibility study	5 healthy participants.	Findings reported in this work indicate that the P300 potential is useful for measuring the individual’s attention level in a BCI video game environment. An additional result was the design and implementation of a new BCI video game.	Participants reported that BCI-game activities helped to stay engaged during the session.
[43]	Engineering journal	Effectiveness study	10 healthy participants (age range 20 to 30).	A BCI video game capable of helping users to improve their attention level, meditation level, and spatial memory by using a dynamic of incremental complexity that requires a higher level of concentration. Results show that after some trials, participants increased their performance in the game from 74% to 98%, while reducing the time they required to reach the level of concentration needed to complete the levels.	BCI system proved to be a tool to improve cognitive skills such as attention level, mediation level, and spatial memory. Also, the results showed that the BCI game helps keep the participants’ attention level during all the tests.
[44]	Engineering proceedings	Effectiveness study	Children living with ADHD (age range 7 to 11).	This study allows the implementation of a BCI system based on multiple video games for training abilities like waiting, planning, following instructions, and achieving objectives. When this study was published, the system was still in the testing phase with children living with ADHD. Preliminary results with this kind of video game dynamics show a decrease in impulsive behavior.	Target out of scope.
[49]	Engineering proceedings	Effectiveness study	26 children living with ADHD (age range 7 to 12). 13 children were assigned to the experimental group, and the rest were assigned to the control group.	After analyzing the results obtained in both the experimental and the control groups, the authors demonstrate that EEG neurofeedback helps to improve cognitive functions in children living with ADHD at the same rate as traditional treatment.	BCI system results showed that participants improved their intelligence performance scores (WISC-III). Also, participants showed an improvement in their attention.
[50]	Medical journal	Effectiveness study	10 children (8 boys and 2 girls aged 7 to 12) in the intervention group and the same distribution in the control group.	Parent- and teacher-rated inattentive score on the ADHD Rating Scale was −3.0 (4.8) for the BCI group and 0.8 (1.3) for the control group.	Results reported in this study show the inattention level of the intervention group decreased slightly more than that of the control group.
[10]	Medical journal	Effectiveness study	44 children diagnosed with ADHD (31 males and 13 females, mean age = 9.81 years in a range of 7.3 to 12.8 years) and 41 children without a diagnosis but displaying similar behavior (33 males and 8 females, mean age = 9.53 years in a range of 7.4 to 12.6 years).	The results of this study provided evidence for the efficacy of using neurofeedback video games through a BCI device for reducing symptom severity in the ADHD and subclinical groups after neurocognitive training.	The Focus Pocus software proved to be useful for training attention problems, aggression, and externalizing. However, participants’ enjoyment and engagement declined across sessions.
[52]	Engineering proceedings	Feasibility study	9 children diagnosed with ADHD, aged 5 to 12 years old.	The results of this study show the feasibility of using neurofeedback video games through low-cost BCI devices for sustained attention training in children living with ADHD.	Game performance data suggest an improvement in the attention self-regulation skill of the children.
[51]	Engineering journal	Effectiveness study	5 children living with ADHD in the first or second grade (all males).	The results reported in this paper indicate that children living with ADHD improved in reading ability, attention span, and behavioral inhibition.	Reading comprehension tests indicate improved reading aloud and reading comprehension after BCI game training. Also, data analysis shows improvements in attention span and decreases in hyperactive behavior over time for all participants.
[53]	Medical journal	Neuroscience study	105 children living with ADHD, aged 8 to 13 years old.	The results reported in this paper suggest that disruption of the anterior cingulate cortex–dopamine interface may underlie the impairments in motivational control observed in childhood ADHD.	Researchers found that the reward positively impacts dopamine-related signals. Also, they observed that the participants were able to keep attention during the sessions.
[54]	Medical journal	Usability study	42 children living with ADHD, aged 8 to 11 years old, with a mean age of 9.4 years.	The usability findings reported in this study indicate positive acceptance of this video game by children living with ADHD. Additionally, researchers obtained recommendations from parents and children to improve the video game.	The system promotes behavioral learning and strategy use in domains of daily life functioning such as time management, planning/organizing, and prosocial skills.
[29]	Engineering journal	Feasibility study	5 healthy males aged 19 to 26 and 4 people living with ADHD (2 males and 2 females aged 18 to 23).	Results reported in this work show that the system obtained 96% and 98% accuracy in classifying the EEG data to detect the correct attention state during trials with healthy people and people living with ADHD, respectively.	Results showed the system could measure attention to detect ADHD during the BCI game sessions.
[23]	Engineering proceedings	Effectiveness study	53 children living with ADHD. 13 females and 40 males (±1.85, mean age = 9.98).	The results reported in this study indicate that 21 out of 53 children living with ADHD had a slight but statistically significant increase in their attention level after using the system for 8 weeks.	40% of children showed a significant increase in their attention level.
[55]	Engineering proceedings	Effectiveness study	16 healthy participants, 8 in the neurofeedback group (3 females and 5 males aged 27 to 32 years old, ±2.4 years, mean age = 29.6) and 8 in the control group (2 females and 6 males aged 24 to 30 years old, ±3.2 years, mean age = 27.1)	The results reported in this work indicate that the neurofeedback game improves the attention threshold of all participants after 5 days of training. However, the amount of threshold increment was different for each participant.	BCI games were shown to be able to improve the attention level and cognitive skills of healthy participants.

## Data Availability

Restrictions apply to the availability of these data. Data were obtained from 36 papers published on PubMed, Engineering Village, IEEE Xplore, and Scopus, respectively.
